# Central cholinergic system mediates working memory deficit induced by anesthesia/surgery in adult mice

**DOI:** 10.1002/brb3.957

**Published:** 2018-03-23

**Authors:** Xiao Zhang, Xuliang Jiang, Lili Huang, Weitian Tian, Xuemei Chen, Xiyao Gu, Weifeng Yu, Jie Tian, Diansan Su

**Affiliations:** ^1^ Department of Anesthesiology Renji Hospital School of Medicine Shanghai Jiaotong University Shanghai China

**Keywords:** anesthesia/surgery, central cholinergic system, working memory

## Abstract

**Background:**

Postoperative cognitive dysfunction (POCD) is consistently associated with increased morbidity and mortality, which has become a major concern of patients and caregivers. Although POCD occurs mainly in aged patients, it happens at any age. Previous studies demonstrated that anesthesia/surgery had no effects on reference memory of adult mice. However, whether it impairs working memory remains unclear. Working memory deficit would result in many deficits of executive function. We hypothesized that anesthesia/surgery impaired the working memory of adult mice and the central cholinergic system was involved.

**Method:**

Tibial fracture internal fixation under the anesthesia of isoflurane was performed in two‐month‐old C57BL/6 mice. Two days later, the spatial reference memory and working memory were measured by a Morris Water Maze (MWM). Donepezil, an inhibitor of acetylcholinesterase (AChE), was administered in another cohort mice for 4 weeks. Then, the working memory was measured by MWM 2 days after anesthesia/surgery. Western blot was used to detect the protein levels of acetylcholine transferase (ChAT), AChE, vesicular acetylcholine transporter (VAChT), and choline transporter (ChT) in the prefrontal cortex (PFC).

**Results:**

We found that anesthesia/surgery had no effects on the reference memory, but it impaired the working memory in adult mice. Meanwhile, we also found that the protein level of ChAT in PFC decreased significantly compared with that in control group. Donepezil pretreatment prevented working memory impairment and the decrease of the protein levels of ChAT induced by anesthesia/surgery.

**Conclusion:**

These results suggest that anesthesia/surgery leads to working memory deficits in adult mice and central cholinergic system impairment is involved.

## 1 INTRODUCTION

Postoperative cognitive dysfunction (POCD) is the deterioration of cognitive function after anesthesia and surgery, especially learning and memory, which may last for days, months, or even years (Bilotta et al., 2010; Knipp et al., 2008; Newman et al., 2001). POCD can markedly impair postoperative recovery and increase morbidity and mortality (Monk et al., 2008; Steinmetz, Christensen, Lund, Lohse, & Rasmussen, 2009). Although aging is considered to be an independent risk factor for POCD (Benson, Ozdemir, Matthews, & Loftus, 2017), it can occur at any age (Krenk, Rasmussen, & Kehlet, 2010). Monk (Monk et al., 2008) documented that at hospital discharge, POCD was present in 36.6% of young (18–39 years), 30.4% of middle‐aged (40–59 years), and 41.4% of elderly patients (60 years or older). However, the results of basic studies are varied. Our previous study demonstrated that anesthesia/surgery had no effects on the reference memory of adult mice measured by the Morris Water Maze (MWM)(Zhao et al., 2016). Other studies obtained similar results (Feng et al., 2013; Wan et al., 2007). Reference memory refers to a long‐term memory that is established by repeated reinforcement of response to the same stimulus (Roberts, Strang, & Macpherson, 2015). In contrast to reference memory, working memory is a type of short‐term memory that corresponds to a critical cognitive domain required for the representation of objects or places during goal‐directed behavior (Bunge, Ochsner, Desmond, Glover, & Gabrieli, 2001). However, whether anesthesia and surgery attenuate the working memory and the mechanism for this process remains unclear.

Accumulated evidences demonstrated that the central cholinergic system is important in the learning and memory (Ferreira‐Vieira, Guimaraes, Silva, & Ribeiro, 2016; Jin, Peng, Wang, Zhang, & Wang, 2017; Patricio, Soares, & Oliveira, 2017). In a previous study (Su et al., 2011), we demonstrated that the impairment of learning and memory induced by isoflurane exposure can be prevented by pretreatment with donepezil, an inhibitor of acetylcholinesterase (AChE). Therefore, we hypothesize that the central cholinergic system mediates working memory deficits induced by anesthesia/surgery in adult mice.

## 2 MATERIAL AND METHODS

### 2.1 Ethics statement

Ethical approval for study (Permit Number: RJ‐20160101) was provided by the Animal Care and Use Committee of Renji Hospital, Shanghai Jiao Tong University School of Medicine, Shanghai, China (Chairman Dr. Huili Dai) on 1 January 2016. All procedures were performed in accordance with the guidelines of the National Institutes of Health (NIH) for animal care (Guide for the Care and Use of Laboratory Animals, Department of Health and Human Services, NIH Publication No. 86‐23, revised 1985). Efforts were undertaken to minimize suffering and the number of animals used.

### 2.2 Animals

Two‐months‐old male C57BL/6J mice were provided by the Animal Research Center of Shanghai Jiaotong University School of Medicine. The animals were housed in standard cages under controlled laboratory conditions (temperature of 22 ± 2°C, 12‐hr light/12‐hr dark cycle) with free access to regular rodent pellets and water. All mice were allowed to adapt to their new environment for 7 days before beginning the experiments.

### 2.3 Tibial fracture fixation

The aseptic open tibial fracture with intramedullary fixation was performed with anesthesia of isoflurane (including 2.0% isoflurane in 0.30 FiO_2_) as previously described (Feng et al., 2017). Briefly, the right hind paw was shaved and disinfected. A median paw incision was then performed, followed by the insertion of a 0.38‐mm pin into the tibial intramedullary canal. The periosteum was then stripped, and an osteotomy was performed. After performing the fracture surgery, we irrigated the wound and the skin was sutured with 5‐0 Vicryl sutures; after that, animals were allowed to recover spontaneously from the anesthesia. During the procedure, temperature was maintained between 36°C and 37°C with the aid of warming pads. A single dose of butorphanol (0.4 mg/kg, s.c.) was administered for analgesia after anesthetic induction and before the skin incision. Learning and memory were measured by MWM 2 days after surgery.

### 2.4 Donepezil pretreatment

To prove the role of the cholinergic system in working memory impairment in the adult mice, donepezil (5 mg/kg, Pfizer, NY) was administered by oral gavage with a feeding needle in 0.4 ml saline every day for 4 weeks in another cohort mice. Tibial fracture fixation was then performed, and the learning and memory were measured by MWM 2 days after surgery.

### 2.5 Morris water maze

Behavioral testing was conducted in the MWM with a circular water maze tank (110 cm in diameter, 30 cm deep), using a computerized video tracking system. The pool was filled to a depth of 30 cm with water made opaque by adding nontoxic white paint. The water temperature was maintained at 23–25°C by a heating pad located beneath the pool. A circular escape platform (10 cm diameter) was submerged 1 cm below the water surface. The pool was surrounded by curtains. The curtains were white and had distinct cues painted on them. For the behavioral test, animals were maintained in the same rearing conditions throughout the test.

#### 2.5.1 Reference memory test

Before the MWM test, mice were individually handled for 2 min each day for 1 week. As described previously (Zhao et al., 2016), four training days were allowed for the reference memory test. The platform was located in the center of the fourth quadrant. In all trials, each mouse was released into the water facing the pool wall from one of four separate quadrants and allowed to swim until it landed on the platform. Once the mouse found the platform, the trial was terminated, and the mouse was allowed to stay on the platform for 15 s. If the mouse failed to find the platform within 60 s, it was gently guided to the platform and allowed to remain on the platform for 15 s. Four trials were conducted per day, separated by a 30–40‐min inter‐trial interval; the platform remained at the same location throughout the test. The amount of time spent finding and mounting the platform (escape latency) and the swimming speed were calculated from the recorded videos using MWM software (Shanghai Jiliang Software Technology Co. Ltd., China).

**Figure 1:**
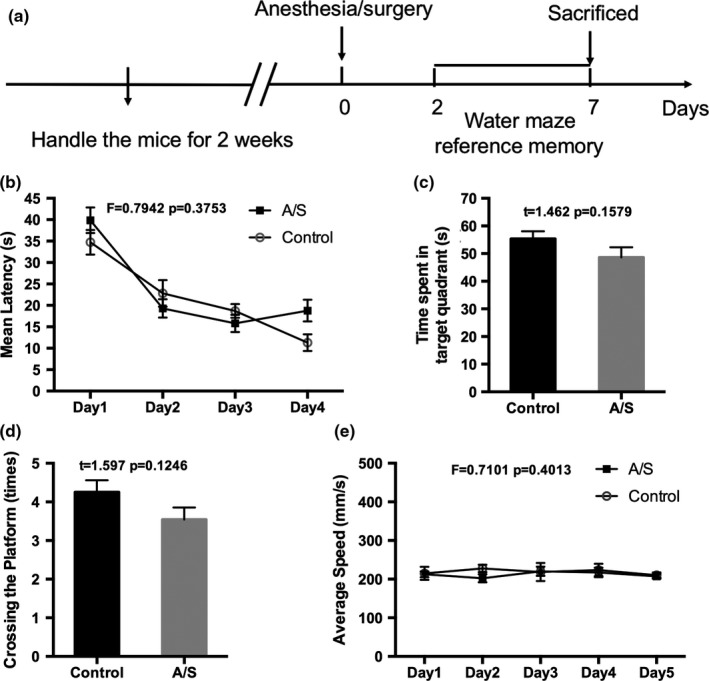
Anesthesia/surgery had no effects on the reference memory in adult mice. (a) Schematic timeline of the experimental paradigm of reference memory; (b) Mean Latency; (c) Swimming time spent in target quadrant (IV) and in the opposite quadrant (II); (d) Times of crossing the platform; (e) Average speed. (*n *=* *12) Data are presented as the mean ± SEM. A/S, Anesthesia/surgery

The probe test was performed on the fifth day of the reference memory test. In this test, the platform was absent, and the animals were allowed to swim freely for 60 s, starting from the quadrant opposite the platform. The times spent in the target and opposite quadrants were recorded (Figure 1a).

#### 2.5.2 Working memory test

**Figure 2:**
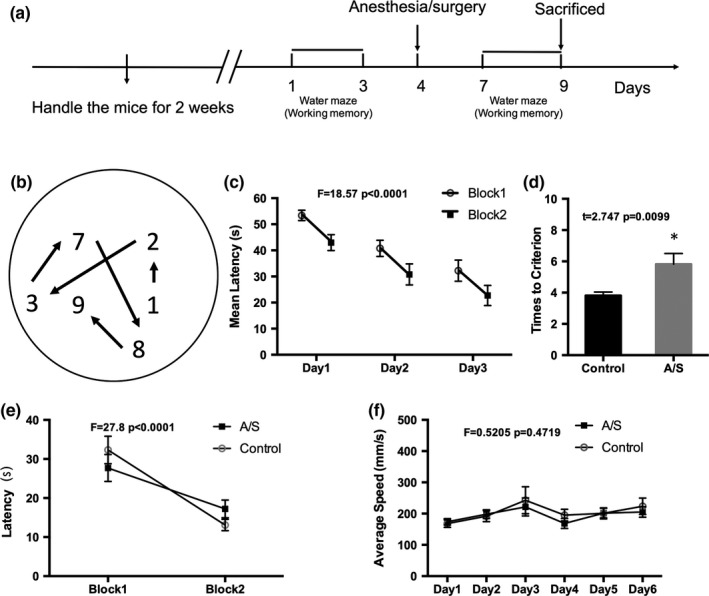
Anesthesia/surgery impaired the working memory in adult mice. (a) Schematic timeline of the experimental paradigm of working memory; (b) Varied platform locations in delayed match‐to‐place task in the MWM. The number represents the day of training or testing; (c) Mean latencies to reach the platform during Block2 was significantly shorter than that of Block 1, which indicated that the mice were able to maintain spatial information across the 30‐min interblock delay after 3 days of training; (d) The number of trials in Block 2 for the mice to reach the criterion of three consecutive trials within 20 s to reach the platform. Mice in the surgery group required significantly more trials to reach criterion; (e) Mean latency in Block 1 and Block 2 on the test day. Latencies declined from Block 1 to Block 2 in mice, which indicating that mice could maintain the spatial information; (f) There was no significantly different between the two groups for the average swimming speed. (*n *=* *15) *: *p *<* *.05 compared with control group. Data are presented as the mean ± SEM. A/S, Anesthesia/surgery

As described previously (Nai et al., 2010), before the MWM test, mice were individually handled for 2 min each day for 1 week. The working memory test lasted for 9 days (Figure 2a). On each training day, mice received six training trials and the platform was in a fixed location (presented in two blocks, three trials in each blocks; interblock interval was 30 min; inter‐trial interval was 15 s). The 30‐min interblock delay was used for the animals to maintain information which was an essential step of working memory. Across training days, the platform position changed for mice to remember a new spatial information each day. For each trial, they were placed into the pool, facing the wall, from one of the starting places. The order of these starting locations was pseudo‐randomly varied throughout training. The trial was complete after the mouse found the platform or 60 s had elapsed. If the mouse failed to find the platform on a given trial, the experimenter guided the mouse onto the platform and the mouse stayed on the platform for 15 s.

For the anesthesia/surgery group, after three training days, the mice received anesthesia/surgery, and rested for 3 days, then continued the fourth and fifth days of training. On the test day, 30 min after Block 1, mice were returned to the water maze for modified Block 2 in which they received multiple trials until they reached a criterion of three consecutive trials, each less than 20 s. In Block 2, continuous trials, starting from different locations (sequence counterbalanced across mice), were performed until the mice reached the criterion. If the mice did not reach the criterion within 10 trials, the test was stopped. This “trial to criterion” index has previously been shown to be a sensitive index of performance (Chen et al., 2000).

### 2.6 Western blot

Animals were sacrificed after MWM, and the brains were harvested. PFC tissue was homogenized with a tissue grinder in RIPA (Beyotime Biotechnology, China), containing a phosphatase and protease inhibitor cocktail, followed by centrifugation of the homogenized tissue samples at 12,000 g for 15 min at 4°C. The supernatants were collected, and their protein concentrations were measured using the Thermo protein assays (Thermo, USA). Samples (20 μg) were electrophoresed on sodium dodecyl sulfate‐polyacrylamide gel electrophoresis gels (Beyotime Biotechnology, China), and the separated proteins were transferred at 300 mA for 70 min onto nitrocellulose membranes (pore size, 0.45 μm, Millipore, USA). The membranes were blocked for 1 hr with Tris‐buffered saline with Tween‐20, containing 3% bovine serum albumin (BSA), followed by incubation with primary antibodies [choline acetyltransferase: ChAT (1:1,000 dilution, number ab181023, abcam, USA); vesicular acetylcholine transporter: VAChT (1:1,000 dilution, number ab134298, abcam, USA); acetylcholinesterase: AChE (1:1,000 dilution, NB1‐59170, NOVUS, USA); high affinity choline transporter: ChT (1:1,000 dilution, number PA5‐42485, Thermo, USA)] and the appropriate secondary antibodies coupled to horseradish peroxidase.

### 2.7 Statistical analysis

Statistical Package for the Social Sciences (SPSS) software, version 20.0, was used for the statistical analyses. The Western blot data were analyzed using independent Student's *t* test. Two‐way ANOVA with repeated‐measures was used to analyze the MWM data, followed by a Bonferroni multiple comparison test.

All data are presented as the mean ± SEM. Differences were considered to be statistically significant at *p *<* *.05.

## 3 RESULTS

### 3.1 Anesthesia/surgery had no effects on the reference memory in adult mice

The spatial reference memory was examined by a Morris Water Maze (MWM) 2 days after tibial fracture internal fixation. The MWM test in adult mice showed that the mean latency of the control group and the surgery group was shortened with the prolongation of the training days, but there was no significant difference between the two groups (Figure 1b). In the probe test, swimming time in the target quadrant (quadrant IV) is much longer than that in the opposite quadrant (quadrant II) for all animals. However, there was no significant difference between the two groups in time spent in the target quadrant and times crossing the platform among the groups (Figure 1c,d). The average swimming speed was not significantly different between the two groups (Figure 1e).

### 3.2 Anesthesia/surgery led to working memory deficits in adult mice

The working memory was examined by a MWM. We trained mice in a delayed match‐to‐place (DMP) memory version of the water maze (Chen et al., 2000). During training, mice learned to navigate to a new escape location each day (Figure 2b). On each day, mice received two blocks of training (three trials each), separated by a 30‐min delay. Following 3 days of training, escape latencies were significantly reduced in the second block compared with the first block (*p *<* *.0001), indicating that the mice were able to maintain spatial information across the 30‐min interblock delay (Figure 2c). On the fourth day, we performed the surgery. After 2 days’ rest, another 2 days’ training follows. On the test day, the latency of Block 1 in each group was higher than that in Block 2 latency (*p *<* *.0001,Figure 2e); however, there was no significant difference between the two groups.

A more sensitive measure in the mouse DMP task, “trials to criterion” was used to further explore the working memory deficits (Chen et al., 2000). We continued training the mice in the DMP task and determined how many trials were required for mice to reach criterion performance (i.e., the number of trials needed to reach three consecutive trials within 20‐s latency). We found that mice in the surgery group required significantly more trials to reach criterion (5.8 ± 0.68) compared with mice in the control group (3.81 ± 0.23, *p *=* *.0099), indicating that anesthesia and surgery impair spatial working memory (Figure 2d). The average swimming speed was not significantly different between the two groups (Figure 2f).

### 3.3 Anesthesia/surgery decreased ChAT protein levels in the PFC

**Figure 3:**
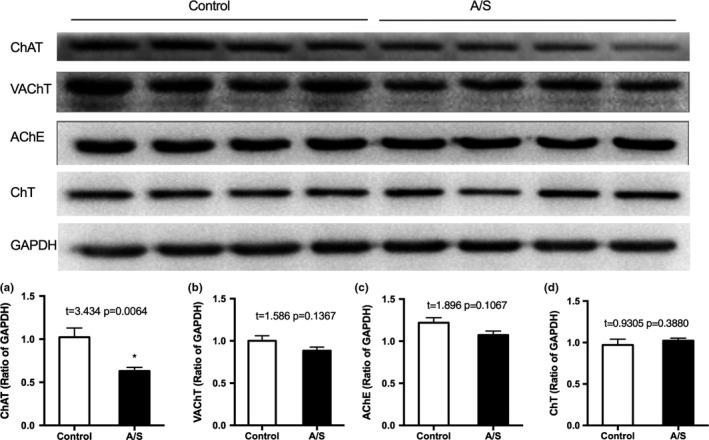
Anesthesia/surgery decreased the ChAT protein levels in the PFC. (a) ChAT; (b) VAChT; (c) AChE; (d) ChT protein levels in the PFC. (*n *=* *6) *: *p *<* *.05 compared with control group. Data are presented as the mean ± SEM. ChAT, choline acetylase; PFC, prefrontal cortex; AChE, acetylcholinesterase; VAChT, vesicular acetylcholine transporter; ChT, choline transporter

Animals were sacrificed after MWM, and the brains were harvested. Western blot was used to detect the protein levels of the markers of cholinergic nerve, acetylcholine transferase (ChAT), acetylcholinesterase (AChE), vesicular acetylcholine transporter (VAChT), and choline transporter (ChT) in the prefrontal cortex (PFC). Western blotting results showed that anesthesia/surgery decreased ChAT protein levels of PFC (*p *=* *.0064). However, there were no significant differences between the two groups with regard to VAChT, AChE, or ChT protein levels (Figure 3).

### 3.4 Donepezil pretreatment prevented working memory impairment and ChAT changes from anesthesia/surgery

**Figure 4:**
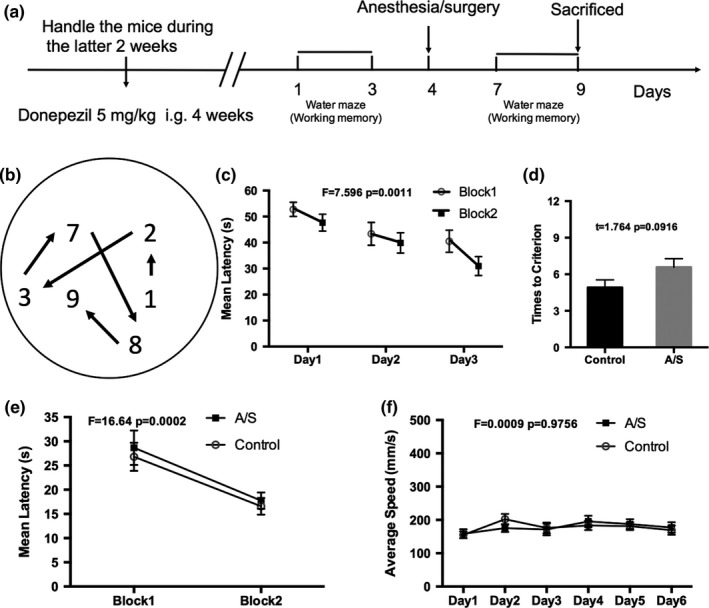
Donepezil pretreatment prevented working memory impairment from anesthesia/surgery. (a) Schematic timeline of the experimental paradigm of working memory; (b) Varied platform locations in delayed match‐to‐place task in the MWM. The number represents the day of training or testing; (c) Mean latencies to reach the platform during Block2 were significantly shorter than that of Block 1, which indicated that the mice were able to maintain spatial information across the 30‐min interblock delay after 3 days of training; (d) The number of trials in Block 2 for the mice to reach the criteria of three consecutive trials within 20 s to reach the platform. There was no significantly difference in trials which both groups need to reach criterion; (e) Mean latency in Block 1 and Block 2 on the test day. Latencies declined from Block 1 to Block 2 in mice, which indicating that mice could maintain the spatial information; (f) There was no significantly different between the two groups for the average swimming speed. (*n *=* *12) Data are presented as the mean ± SEM. A/S, Anesthesia/surgery

To prove the role of the cholinergic system in working memory impairment in the adult mice, donepezil, an inhibitor of AChE, was administered. After 4 weeks of pretreatment with donepezil (5 mg/kg, Pfizer, NY) by oral gavage, the MWM was conducted to measure working memory. As shown in Figure 4c, following 3 days of training, escape latencies were significantly reduced in Block 2 compared with Block 1 (*p *=* *.0011), indicating that the mice were able to maintain spatial information across the 30‐min interblock delay. On the test day, the latency of Block 1 in each group was higher than that in Block 2 latency (*p *<* *.0001,Figure 4e), but there was no significant difference between the two groups. In the DMP task, there was no significant difference in the trials that both groups needed to reach criterion, indicating that donepezil pretreatment prevented working memory impairment from anesthesia and surgery (Figure 4d). The average swimming speed was not significantly different between the two groups (Figure 4f).

**Figure 5:**
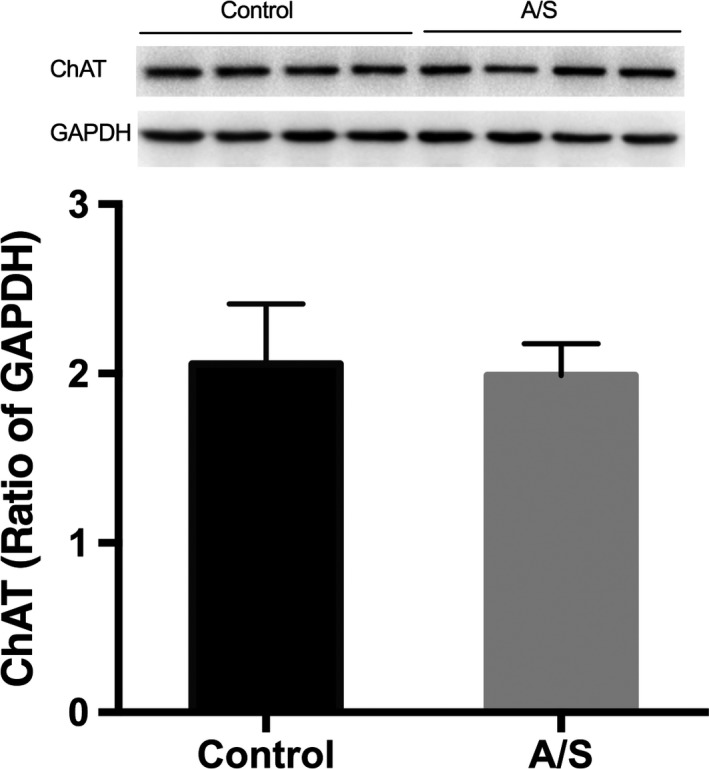
No ChAT protein levels changes were observed after donepezil pretreatment. There was no significant difference between the two group for the protein level of ChAT in PFC after pretreatment with donepezil, an inhibitor of AChE. (*n *=* *6). Data are presented as the mean ± SEM. ChAT, choline acetylase; PFC, prefrontal cortex; AChE, acetylcholinesterase; Donepezil pretreatment

Western blotting results showed that ChAT protein levels did not change in the donepezil+surgery group compared to the donepezil group (Figure 5), which suggests that donepezil pretreatment could prevent the decrease of ChAT protein levels caused by anesthesia/surgery.

## 4 DISCUSSION

The present study demonstrated that anesthesia/surgery impaired working memory but not reference memory in adult mice. The central cholinergic system was involved and donepezil, an inhibitor of AChE, pretreatment prevented the working memory impairment after anesthesia/surgery.

Tibial fracture fixation is one typical mice model of POCD. Maze's group reported this model initially in 2010 (Cibelli et al., 2010). Splenectomy (Lu et al., 2015), partial hepatectomy (Tian et al., 2017), exploratory laparotomy (Qiu et al., 2016), and right common carotid artery occlude (Sanches et al., 2013) were also utilized as POCD model. Maze group demonstrated that tibial fracture fixation impaired the performance of mice in the fear conditioning test (Cibelli et al., 2010). To our knowledge, this is the first study to demonstrate working memory impairment after anesthesia/surgery in adult mice.

Working memory decline is one of the main concerns of POCD. Research from Sebastian Holinski (Holinski et al., 2011), and Kyriakos Anastasiadis, et al.(Anastasiadis et al., 2011), demonstrated that working memory dysfunction occurred in patients after cardiac surgery. Yin et al. (Yin, Wang, & Liu, 2014) showed that propofol impairs short‐term memory following elective hernia surgery in children between 7 and 13 years of age. Working memory is responsible for temporary storage of information and is required to support ongoing everyday activities such as instruction following, mental arithmetic, reasoning, spatial visualization, and problem‐solving (Dunning, Holmes, & Gathercole, 2013). Clinical trials have shown that young people with poor working memory skills in school are at high risk of educational underachievement in reading and mathematics (De Weerdt, Desoete, & Roeyers, 2013; Gathercole, Brown, & Pickering, 2003), and typically make poor progress in all assessed areas of the academic curriculum (Geary, 2004). Working memory is important in young people and may have influence on their daily life.

Central cholinergic system impairment was involved in working memory deficits induced by anesthesia/surgery. The cholinergic system has been implicated in many aspects of cognition including the partitioning of attentional resources, working memory, inhibition of irrelevant information, and improved performance on effort‐demanding tasks (Dumas & Newhouse, 2011; Newhouse, Potter, Kelton, & Corwin, 2001). Beninger RJ, et al. (Beninger, Wirsching, Jhamandas, Boegman, & el‐Defrawy, 1986) observed that after treatment with kainite i.p., the rats’ cortical ChAT decreased; the rats exhibited worse performance on working memory tasks than the sham group, indicating that cholinergic neurons of the basocortical system were involved in working memory. A recent study from Markett et al. (Markett, Montag, & Reuter, 2010) reported that the nicotinic acetylcholine receptor gene CHRNA4 was involved in visuospatial working memory capacity. The prefrontal cortex (PFC) has been found to be essential for working memory processes (Dunning et al., 2013). Interestingly, in our study, we found the protein level of ChAT significantly decreased in PFC after tibial fracture fixation, suggesting that central cholinergic system impairment was involved in working memory deficits induced by anesthesia/surgery.

In present study, we demonstrated that donepezil pretreatment could improve the performance of working memory in adult mice after anesthesia and surgery, which showed similar results to Ruifeng Liu's findings in adult monkeys (Liu et al., 2017). Donepezil is a medicine approved by the FDA and could improve cognition for the patients with Alzheimer's disease and stroke traumatic brain injury and even for normal older adults (Berthier, Hinojosa, Martin Mdel, & Fernandez, 2003; Black et al., 2007; Yesavage et al., 2002; Zhang, Plotkin, Wang, Sandel, & Lee, 2004). And our study provides experimental evidence that donepezil may have translational potential for POCD prevention and treatment (Su et al., 2011). However, 4 weeks pretreatment, as utilized in the present study, is too long to carry out in clinics. A study is currently conducted in our laboratory to optimize the pretreat time of donepezil.

Neuro‐inflammation (Xu et al., 2014a), Aβ accumulation (Xie et al., 2006), and Tau phosphorylation (Liu et al., 2012) are now considered relating to the development of POCD. Central cholinergic system has inhibitory effects on neuro‐inflammation through a cholinergic anti‐inflammation reflex (Tyagi, Agrawal, Nath, & Shukla, 2010). It is possible that the impaired cholinergic system cannot inhibit the neuro‐inflammation effectively, which might contribute to the development of POCD. More studies are warranted to investigate the hypothesis. Aβ accumulation and Tau phosphorylation are also widely accepted as mechanisms of POCD (Xie et al., 2013; Xu et al., 2014a,b; Zhang et al., 2013). Actually, increased Aβ and Tau phosphorylation would damage cholinergic neuron in the basal forebrain, reduce cortical AChE‐positive fiber density, and impair the learning and memory (Harkany et al., 1998; O'Mahony et al., 1998). Further studies are needed to better clarify the relationship among Aβ accumulation, Tau phosphorylation, and changes in central cholinergic system after anesthesia/surgery.

Zhang et al. demonstrated earlier that pain induced by surgery contributes to the learning and memory impairment after anesthesia/surgery (Zhang et al., 2013). In current study, as butorphanol was utilized to prevent postoperative pain and no animals displayed any signs of pain (i.e., writhing, scratching or biting), the possible contribution to POCD from postoperative pain can thus be excluded.

Our study has several limitations. Firstly, we did not have an anesthesia only group, so we cannot tell the effects of anesthesia *per se* on the working memory. Instead, we viewed anesthesia and surgery as a whole, as in clinical settings, anesthesia always accompanies surgery. Secondly, we did not observe the cognition changes in a long‐term follow‐up. We therefore do not know how long the working memory deficit persists. And thirdly, mechanisms underlying why anesthesia/surgery could impair the central cholinergic nervous system and why cholinergic deficiency leads to POCD remain to be explored. Previous studies indicate that synaptic dysfunction (VanGuilder et al., 2011) is directly connected with cognitive impairment. Further investigations are needed to determine the connection of cholinergic deficiency and synaptic dysfunction following anesthesia and surgery.

## 5 CONCLUSIONS

Anesthesia/surgery impaired the working memory but not the reference memory in adult mice, and the central cholinergic system was involved. Donepezil, an inhibitor of AChE, might be helpful to reduce the working memory deficit after anesthesia/surgery in adult mice. Although inspiring, present results were obtained from animal studies, which cannot be applied directly to clinical settings. Clinical trials are warranted for the application of donepezil to prevent POCD in patients.
